# Differential gene expression profile between progressive and de novo muscle invasive bladder cancer and its prognostic implication

**DOI:** 10.1038/s41598-021-85137-1

**Published:** 2021-03-17

**Authors:** Raquel Carrasco, Laura Izquierdo, Antoine G. van der Heijden, Juan José Lozano, Marco Franco, Mercedes Ingelmo-Torres, Fiorella L. Roldan, Montserrat Llorens, María José Ribal, Lourdes Mengual, Antonio Alcaraz

**Affiliations:** 1 Laboratory and Department of Urology, Hospital Clínic, Institut d’Investigacions Biomèdiques August Pi i Sunyer (IDIBAPS), Universitat de Barcelona, Barcelona, Spain; 2 Department of Urology, Radboud University Medical Center, Nijmegen, The Netherlands; 3 CIBERehd, Plataforma de Bioinformática, Centro de Investigación Biomédica en red de Enfermedades Hepáticas y Digestivas, Barcelona, Spain; 4 Laboratory of Urology, Hospital Clínic de Barcelona, Centre de Recerca Biomèdica CELLEX, office B22, C/Casanova, 143, 08036 Barcelona, Spain

**Keywords:** Prognostic markers, Translational research, Bladder cancer, Gene expression analysis

## Abstract

This study aimed to ascertain gene expression profile differences between progressive muscle-invasive bladder cancer (MIBC) and de novo MIBC, and to identify prognostic biomarkers to improve patients’ treatment. Retrospective multicenter study in which 212 MIBC patients who underwent radical cystectomy between 2000 and 2019 were included. Gene expression profiles were determined in 26 samples using Illumina microarrays. The expression levels of 94 genes were studied by quantitative PCR in an independent set of 186 MIBC patients. In a median follow-up of 16 months, 46.7% patients developed tumor progression after cystectomy. In our series, progressive MIBC patients show a worse tumor progression (p = 0.024) and cancer-specific survival (CSS) (p = 0.049) than the de novo group. A total of 480 genes were found to be differently expressed between both groups. Differential expression of 24 out of the 94 selected genes was found in an independent cohort. *RBPMC2* and *DSC3* were found as independent prognostic biomarkers of tumor progression and *CALD1* and *LCOR* were identified as prognostic biomarkers of CSS between both groups. In conclusion, progressive and de novo MIBC patients show different clinical outcome and gene expression profiles. Gene expression patterns may contribute to predict high-risk of progression to distant metastasis or CSS.

## Introduction

At diagnosis, 75% is non-muscle-invasive bladder cancer (NMIBC) and 25% is muscle-invasive bladder cancer (MIBC) or metastatic^1^. NMIBC and MIBC show differences in terms of prognosis and treatment^2^. However, high-grade NMIBC invades deep into the lamina propria and share morphologic, clinical and molecular characteristics with MIBC, including aggressive behavior and lethality potential^3,4^. Therefore, despite proper NMIBC management, about 40–50% of high grade non-invasive tumors will progress to MIBC during follow-up^5^.

Both, progressive and de novo MIBC are treated with radical cystectomy (RC), but a different clinical outcome has been described between both MIBC groups after RC. Several studies have shown that progressive MIBC had a significantly worse prognosis compared to de novo invasive tumors^6,7^, although it remains a controversial issue. Chen et al.^8^ did not find survival differences between progressive and de novo MIBC groups in a set of 4102 patients. Contrarily, more recently, Peng Ge et al.^9^ demonstrated survival differences between both groups in a series of 4075 MIBC patients, where progressive MIBC patients had a worse cancer specific survival (CSS) than de novo group. These survival differences may indicate that progressive MIBC harbor genetic characteristics which confer them with a more aggressive conduct than de novo MIBC. Therefore, both MIBC groups could represent different molecular entities and biological behavior. In a recent study, Pietzak et al.^10^ demonstrated that progressive MIBC had lower response rates to neoadjuvant chemotherapy compared with de novo MIBC, also suggesting genetic differences between both MIBC groups.

Currently, there is no data regarding the molecular features of progressive and de novo MIBC and therefore, the risk of progression is determined according to clinicopathological parameters defined European Association of Urology (EAU) or American Urological Association (AUA) urology guidelines^11,12^. However, these clinicopathological parameters are not accurate enough to stratify those patients that will develop tumor progression from those who will not, and patients with high-risk progression tumors may obtain an inappropriate treatment^9^.

Here, we examine gene expression patterns of progressive and de novo MIBC to elucidate genetic differences between them and the molecular pathways that lead to tumor progression, and to identify prognostic biomarkers that could help us to tailor treatment in MIBC patients.

## Results

### Clinicopathological variable comparison between progressive and de novo MIBC

A total of 212 MIBC patients (104 progressive and 108 de novo) were included in this study (Table 1). In a median follow-up of 16 months, 99 (46.7%) patients progressed. Of them, 56 (26.4%) were from the progressive group [23 with LN(+)] and 43 (20.3%) from de novo group [19 with LN(+)]. The median time to progression was 8 months (8 and 9 months for progressive and de novo patients, respectively). All progressive MIBC patients who were treated with neoadjuvant chemotherapy (n = 6) progressed. On the other hand, 12 (41%) of de novo MIBC patients progressed after neoadjuvant chemotherapy treatment.

**Table 1:** Demographic and clinicopathological characteristics of enrolled patients.

	Overall		Discovery phase		Validation phase	
	De novo	Progressive	De novo	Progressive	De novo	Progressive
	MIBC	MIBC	MIBC	MIBC	MIBC	MIBC
	*N (%)*	*N (%)*	*N (%)*	*N (%)*	*N (%)*	*N (%)*
Overall	108	104	12	14	96	90
**Gender**						
Male	90 (83.3)	69 (66.3)	12 (100)	13 (92.9)	78 (81.2)	56 (62.2)
Female	18 (16.7)	35 (33.7)	–	1 (7.1)	18 (18.8)	34 (37.8)
Median age (years)	69	72	66	74	72	71
**Pathological stage**						
pT0–T1	28 (26)	11 (10.6)	8 (66.6)	1 (7.1)	20 (20.8)	10 (11.1)
pT2	50 (46.3)	46 (44.2)	–	9 (64.3)	50 (52.1)	38 (42.2)
pT3	20 (18.5)	29 (27.9)	2 (16.7)	3 (21.5)	18 (18.8)	26 (28.9)
pT4	10 (9.2)	18 (17.3)	2 (16.7)	1 (7.1)	8 (8.3)	16 (17.8)
Carcinoma in situ (CIS)	36 (33.3)	20 (19.2)	7 (58.3)	3 (21.5)	29 (30.2)	17 (18.9)
**Lymph nodes (LN)**						
LN(+)	20 (18.5)	30 (28.8)	3 (25)	–	17 (17.7)	30 (33.3)
LN(−)	88 (81.5)	74 (71.2)	9 (75)	14 (100)	79 (82.3)	60 (66.7)
Neoadjuvant Chemotherapy	29 (26.8)	6 (5.6)	10 (83.3)	–	19 (19.8)	6 (6.7)
Median TURBT Number	–	3	–	3	–	2.5
**Last NMIBC stage**^**a**^					–	
T0	–	7 (6.7)	–	2 (14.3)	–	5 (5.6)
Ta	–	23 (22.1)	–	3 (21.5)	–	20 (22.2)
T1	–	49 (47.1)	–	8 (57.1)	–	41 (45.6)
CIS	–	11 (10.6)	–	1 (7.1)	–	10 (11.1)

^a^Last NMIBC stage was not available in 14 progressive patients.

**Figure 1:**
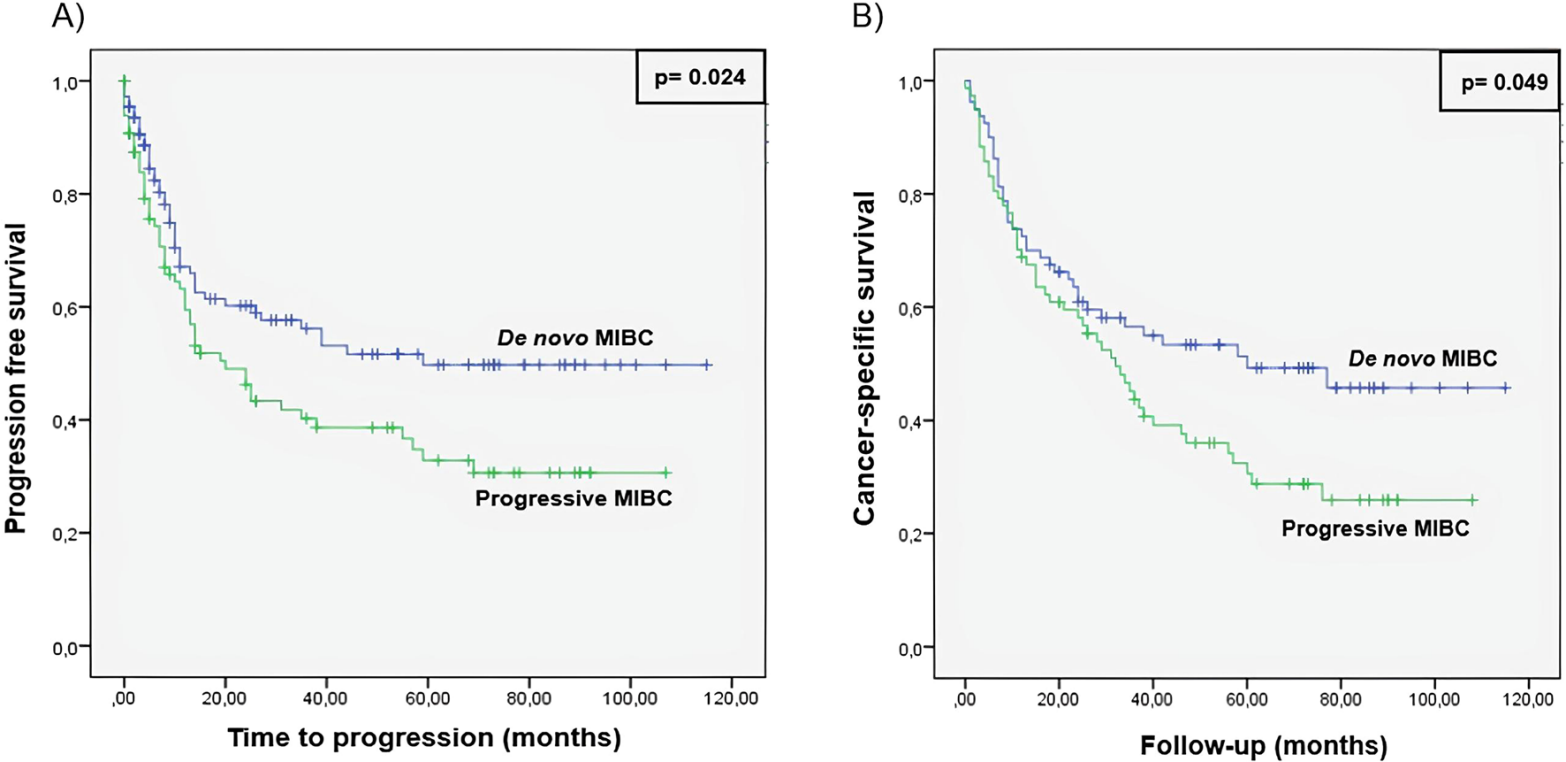
Kaplan–Meier curve for (**A**) tumor progression and (**B**) cancer specific survival comparing progressive and de novo MIBC groups.

During the follow-up, 144 (67.9%) patients died; 87 (41%) of them due to cancer progression [51 (24%) from progressive (21 with LN (+)) and 36 (17%) from de novo (15 with LN(+)) group]. The median time of CSS was 29 months (26 and 30 months for progressive and de novo patients, respectively). Progressive patients showed a significant difference in terms of survival compared with de novo patients (Fig. 1).

### Discovery phase: gene expression patterns comparison between progressive and de novo MIBC

**Figure 2:**
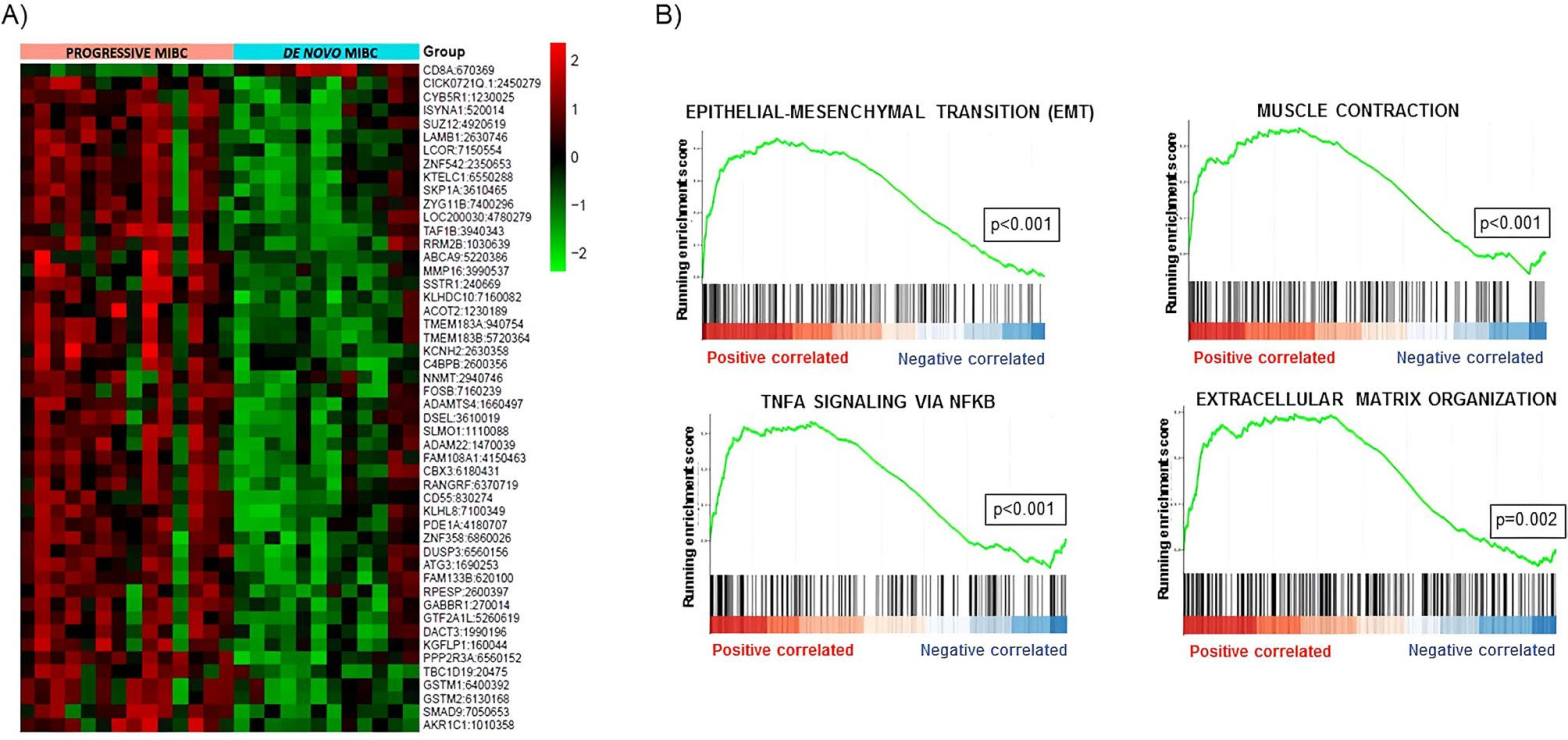
Gene expression profiles in progressive and de novo MIBC samples. (**A**) Heat map displaying the 50 most differently expressed genes between progressive and de novo MIBC samples. Red pixels correspond to an increased abundance of mRNA in the samples, whereas green pixels indicate decreased mRNA levels. Rows represent genes and columns represent experimental samples. (**B**) Gene set enrichment analysis (GSEA) shows positive correlation of overexpressed genes in pathways involved in tumor progression.

The analysis of 26 MIBC patients (Table 1) using Illumina microarray resulted in the identification of 480 differently expressed transcripts between progressive and de novo MIBC groups (Supplementary Table S1); 468 up-regulated and 12 down-regulated in progressive compared with de novo MIBC patients. Heat map based on the 50 most differently expressed genes shows a clear distinction between both groups (Fig. 2A). GSEA based on Hallmark, KEGG and Reactome databases identified that overexpressed genes were positively enriched in pathways such as epithelial-mesenchymal transition (EMT), muscle-contraction, regulation of NFKB in response to TNFA, and extracellular matrix organization (Fig. 2B).

### Gene expression validation

A total of 94 genes were analyzed by qPCR in a cohort of 186 MIBC patients (Table 1). Twenty-four out of 94 genes were found to be differently expressed between progressive and de novo MIBC samples (Table 2). Of note, all genes were overexpressed in progressive compared with de novo MIBC patients. The *USP42* gene has a different sense of differential expression in microarray and qPCR experiments, therefore this gene cannot be considered. Using the remaining 23 genes, a network using GeneMANIA was generated. We found that there is co-expression and physical interactions between the vast majority of these 23 DEGs (Supplementary Fig. S1A). Moreover, several pathways were significantly enriched by this gene expression profile such as focal adhesion, PTK6 expression, muscle contraction and proteoglycans in cancer (Supplementary Fig. S1B).

**Table 2:** Significant DEGs between progressive and de novo MIBC of validation phase (RT-qPCR) and comparison with fold change in genes from the discovery phase (Microarray).

Gene	Source	Validation phase		Discovery phase	
		FDR	Fold change	p-Value	Fold change
*ABCC9*	Microarray	0.04	3.41	< 0.001	4.10
*ARL5A*	Microarray	0.001	1.49	0.001	2.47
*CALD1*	Microarray	< 0.001	3.34	< 0.001	3.90
*FLNC*	Microarray	< 0.001	4.48	0.001	4.83
*GEM*	Microarray	0.004	3.13	0.003	3.06
*HIF1A*	Microarray	< 0.001	1.99	< 0.001	3.30
*RGS2*	Microarray	0.001	4.54	< 0.001	4.25
*SMAD5*	Microarray	0.001	1.84	< 0.001	2.82
*USP42*	Microarray	0.001	2.23	< 0.001	− 5.30
*ITGA5*	Microarray	< 0.001	3.68	< 0.001	4.26
*CD44*	Literature	< 0.001	2.98	0.071	− 1.48
*PDCD1LG2*	Literature	0.004	2.26	0.814	1.09
*IDO1*	Literature	0.02	2.31	0.579	− 1.16
*PGM5*	Literature	< 0.001	5.40	0.308	1.57
*DES*	Literature	< 0.001	7.68	0.779	1.16
*C7*	Literature	< 0.001	4.77	0.067	2.09
*SFRP4*	Literature	< 0.001	5.28	0.119	2.45
*COMP*	Literature	< 0.001	3.60	0.752	1.21
*ZEB1*	Literature	< 0.001	3.33	0.071	1.59
*ZEB2*	Literature	< 0.001	2.83	0.210	1.32
*TWIST1*	Literature	0.003	8.22	0.886	1.04
*MSN*	Literature	0.001	1.94	0.834	− 1.07
*NR3C1*	Literature	0.001	1.97	0.330	1.21
*CTSE*	Literature	0.007	2.35	0.238	1.66

The source from what the gene was taken (microarray/literature) is also shown.

*FDR* False Discovery Rate.

### Survival analysis

Since we found that progressive and de novo MIBC show different gene expression patterns, survival analysis was performed independently in each of the groups. Additionally, only LN(−) patients were evaluated in this analysis, since lymph node status is unknown at the time of TURB. Furthermore, LN(+) patients had significantly worse outcomes than LN(−) patients (Supplementary Fig. S2), which could introduce a bias in the analysis. Thus, 132 patients were included in this analysis (55 progressive and 77 de novo).

Clinical and molecular variables were evaluated by Cox regression analysis (Tables 3, 4). Univariate and multivariate regression analysis for tumor progression showed that expression of *RBPMS2* and *DSC3* were found to be independent prognostic biomarkers in progressive and de novo MIBC groups, respectively. Furthermore, univariate and multivariate regression analysis for CSS showed that *CALD1* and *LCOR* were found to be independent prognostic biomarkers in progressive and de novo MIBC groups, respectively.

**Table 3:** Tumor progression in progressive and de novo MIBC patients.

	Univariate analysis		Multivariate analysis	
	HR (95% CI)	p-value	HR (95% CI)	p-value
**(A) Progressive MIBC**				
*FLNC*	2.73 (1.06–7.05)	0.038	–	0.503
*FGFR1*	2.93 (1.14–7.56)	0.026	–	0.150
*RBPMS2*	3.97 (1.23–12.75)	0.021	8.73 (1.72–44.23)	0.009*
Pathological stage	1.50 (1.07–2.09)	0.017	–	0.332
**(B) De novo MIBC**				
*ITGA5*	2.61 (1.17–5.83)	0.019	–	0.110
*DSC3*	0.21 (0.08–0.55)	0.001	0.20 (0.05–0.75)	0.017*
*MSI1*	2.97 (1.08–8.15)	0.034	–	0.490
*PGM5*	2.51 (1.03–6.10)	0.042	–	0.056
*SNAI1*	2.56 (1.02–6.42)	0.046	–	0.727

*HR* Hazard ratio, *CI* confidence interval.

*Statistically significant values are in bold.

**Table 4:** Cancer-specific survival in progressive and de novo MIBC patients.

	Univariate analysis		Multivariate analysis	
	HR (95% CI)	p-value	HR (95% CI)	p-value
**(A) Progressive MIBC**				
*CALD1*	2.62 (0.99–6.93)	0.044	6.24 (1.37–28.40)	0.018*
Pathological stage	1.43 (0.98–2.09)	0.049	-	0.060
**(B) De novo MIBC**				
*CALD1*	2.40 (1.01–5.73)	0.048	–	0.738
*LCOR*	3.16 (1.30–7.66)	0.011	6.75 (2.03–22.42)	0.002*
*ITGA5*	2.55 (1.09–5.99)	0.031	–	0.206
*DSC3*	0.23 (0.08–0.64)	0.005	–	0.371
*MSI1*	3.19 (1.04–9.78)	0.042	–	0.355

*HR* Hazard ratio, *CI* confidence interval.

*Statistically significant values are in bold..

**Figure 3:**
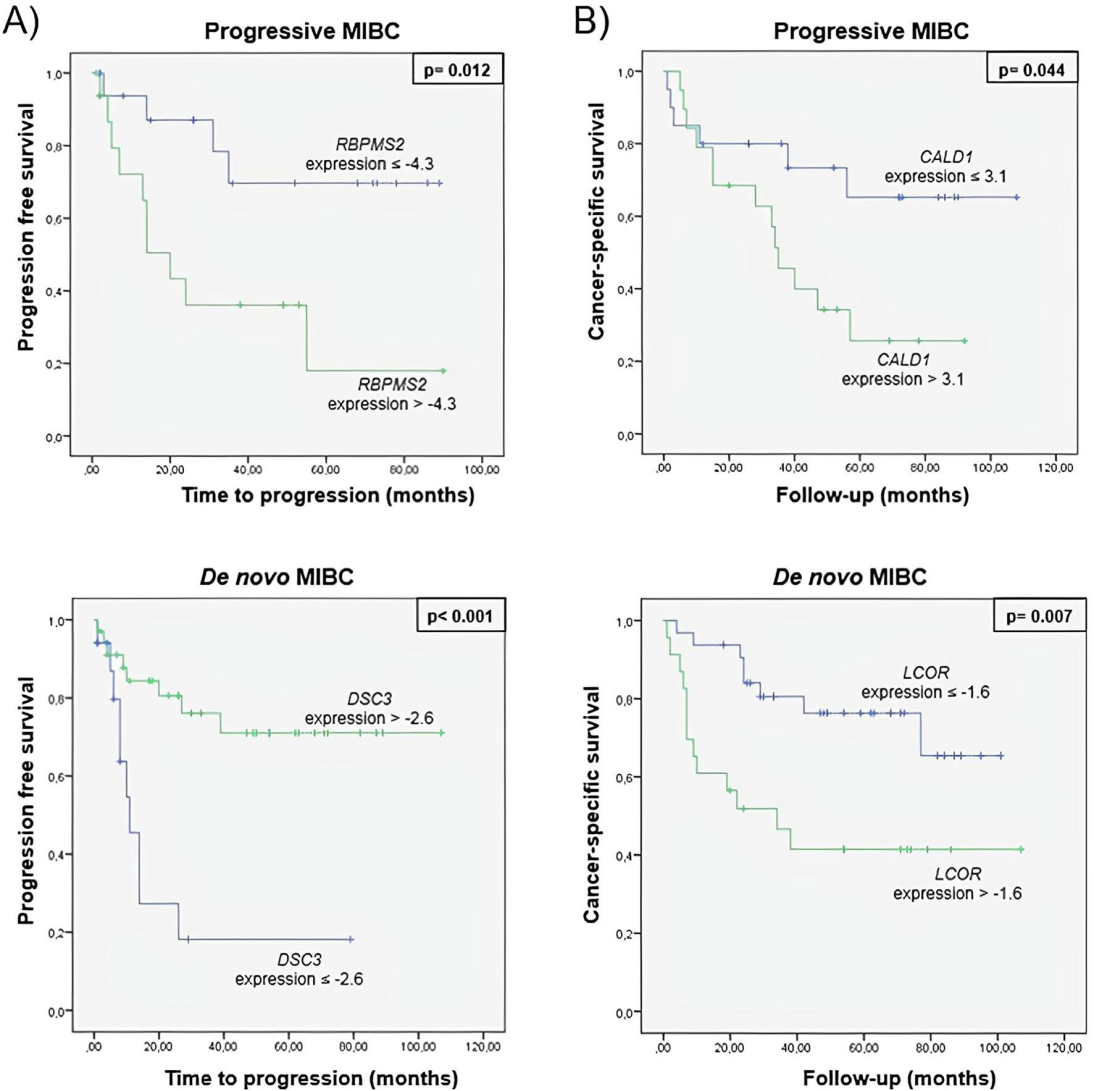
Kaplan–Meier curves for (**A**) tumor progression and (**B**) cancer specific survival of prognostic biomarkers in progressive and de novo MIBC groups.

Thereafter, the median expression value of each prognostic biomarker was used as a cut-off point to classify patients into high-risk and low-risk groups for tumor progression and CSS. Figure 3 shows the Kaplan–Meier curves of the biomarkers in progressive and de novo MIBC patients generated using the selected cut-off point. As shown, expression values were able to discriminate between two groups of MIBC patients with a significant different probability of tumor progression and CSS.

## Discussion

Several studies have reported that patients with MIBC with a previous history of a non-invasive tumor present a worse outcome compared with de novo MIBC patients after RC^6,7,9^. However, these studies are only based on the clinicopathological characteristics of the tumor and no data regarding different molecular features of progressive and de novo MIBC are reported in the literature to date. Here, we have characterized gene expression profiles of progressive and de novo MIBC. Furthermore, we identified prognostic markers in each group of MIBC patients that may contribute to tailor treatment strategies.

Our series corroborates that progressive patients have a worse prognosis than de novo MIBC, as previously reported^6,7,10^. Furthermore, all progressive MIBC patients who were treated with neoadjuvant chemotherapy progressed to metastasis, confirming that these patients have a worse response to this therapy than de novo MIBC patients^10^. The more aggressive behavior of progressive MIBC could be explained by two hypotheses. First, intravesical and cytotoxic cancer therapies administered to NMIBC would induce selection of resistant clones which could play an important role in tumor progression. Second, TURBT could promote the intravesical and hematogenous spread of tumor cells^9^. This is supported by the detection of circulating tumor cells in NMIBC patients after, but not before TURBT^13^. On the contrary, this more aggressive pattern of progressive MIBC could be simply explained by late muscle invasive tumor diagnosis and delayed radical cystectomy^14^. Therefore, NMIBC patients with high-risk of progression to muscle invasion may be considered for an early cystectomy. In fact, early cystectomy is already performed in some high-grade NMIBC selected patients according to urologic guidelines in several centers, including ours^12,15–17^.

To the best of our knowledge, this is the first report demonstrating that progressive and de novo MIBC present distinct molecular signatures. Moreover, we have found that genes overexpressed in progressive MIBC are involved in pathways such as EMT, muscle contraction, TNFA signaling and extracellular matrix organization. All these pathways promote tumor progression and invasion^18–21^, corroborating that gene expression differences between progressive and de novo MIBC may account for the different clinical outcome of these patients. Validation of a subset of these DEGs in an independent and larger cohort further supported data from discovery phase. We found that validated genes are involved in pathways like focal adhesion, *PTK6* expression, muscle contraction and proteoglycans in cancer. Focal adhesion is an essential step in cell migration and its dysregulation promotes cell invasion and metastasis^22^; PTK6 is a protein that regulates normal cell growth, but in tumors it contributes to cell proliferation by sensitizing cells to mitogenic signals^23^; cell contraction and motility is regulated by caldesmon, a component of cytoskeleton in muscle cells^19^, suggesting that up-regulation of *CALD1* in progressive patients could promote cell motility and invasion. Eventually, proteoglycans are key macromolecules that contribute to proliferation, angiogenesis and metastasis, promoting cancer progress^24^. Therefore, these pathways play a crucial role in tumor migration and invasion, promoting EMT^18,25^.

Finally, we have been able to identify prognostic biomarkers to predict the clinical outcome in each group of MIBC patients. We found that *RBPMS2* and *DSC3* are prognostic biomarkers for tumor progression in progressive and de novo MIBC, respectively. Over-expression of *RBPMS2*, a protein involved in the regulation of muscle cell differentiation and proliferation^26^, and down-regulation of *DSC3*, a member of the cadherin family implicated in cell–cell adhesion, have been found in other solid tumors, according our results. *DSC3* has also been previously described as a prognostic biomarker in various solid tumors^27–29^.

On the other hand, we found *CALD1* and *LCOR* as prognostic biomarkers for CSS in progressive and de novo MIBC, respectively. According to our results, over-expression of *CALD1*, a protein that regulates cell motility, and *LCOR*, a protein that modulates expression of the estrogen receptor, has been previously associated with poor prognosis in bladder and other solid tumors^30,31^.

The relevance of the present work falls on the fact that it is the first report to describe molecular differences between progressive and de novo MIBC in a balanced and multicentric cohort. The methodology used to analyze these biomarkers is widely available, reasonably simple and inexpensive, and thus they could be easily implemented in clinical practice. Consequently, gene expression of MIBC could be easily detected from TURBT samples and those patients with a high-risk of progression to distant metastasis or cancer specific mortality could benefit from early adjuvant treatments.

However, we have to acknowledge some limitations. Given the heterogeneity of bladder cancer, one limitation of this study is that we have sampled only one segment of the tumor. Detection of gene expression in liquid biopsy samples could overcome this limitation. In addition, patients with LN(+) have been excluded from survival analysis due to the increased risk of progression of LN(+) patients, decreasing sample size. Therefore, the study has a limited size which can limits the statistical power of the study. A final validation of the results in a larger, independent series is necessary to define the real role of these biomarkers and for their clinical implementation.

## Conclusions

Progressive and de novo MIBC patients show different gene expression profiles. Progressive patients show overexpression of genes involved in tumor invasion and migration, resulting in a worse prognosis of these patients compared with the de novo MIBC group. Progressive and de novo MIBC groups present different prognostic biomarkers for tumor progression (*RBPMS2* in progressive MIBC and *DSC3* in de novo MIBC) and for CSS (*CALD1* in progressive MIBC and *LCOR* in de novo MIBC). These biomarkers may contribute to predict high-risk of progression to distant metastasis or cancer specific mortality and consequently, to tailor treatment and surveillance strategies in these patients.

## Materials and methods

### Patients and samples

Retrospective multicenter study including 212 patients (median age 72 (range 37–100) years; 159 males, 53 females) with MIBC who underwent radical cystectomy with lymphadenectomy in two different centers (Hospital Clinic, Barcelona, Spain and Radboud University Medical Center, Nijmegen, The Netherlands) between 2000 and 2019. Two groups were formed: progressive MIBC, patients with primarily NMIBC who showed progression to MIBC (N = 104) and de novo MIBC, patients with primarily MIBC (N = 108) (Table 1).

Samples were obtained either from transurethral resection of the bladder tumor (TURBT; N = 132) showing muscle-invasive disease or from cystectomy specimens (N = 80). At the moment of cystectomy, 50 MIBC patients had positive lymph nodes [LN(+)]. None of the patients had distant metastasis.

The work was approved by the Ethical Committee of the Clinical Investigation from Hospital Clinic, Barcelona, Spain. All the procedures were carried out in accordance with the relevant guidelines and regulations of CPMP/ICH/135/95. All tissue samples were obtained under an institutional review board-approved protocol and written informed consent was obtained from all participants.

Postoperative follow-up was in the first year 3-monthly and in the second and third year biannual. After 3 years disease-free, patients were followed up yearly. In the follow-up abdominal and/or pelvic CT scans were performed. Tumors were considered progressive in case of local (relapse) or distant metastasis. Five patients were lost of follow-up.

### Tissue specimens and RNA isolation

Formalin-fixed paraffin-embedded (FFPE) tissue sections of 20 µm were obtained from IDIBAPS and Radboud University Medical Center tumor biobanks, without direct involvement of human participants**.** Total RNA was isolated from FFPE sections using the RecoverAll Total Nucleic Acid Isolation Kit (Ambion, Inc. Austin, TX, USA), according to the manufacturer’s instructions. RNA was quantified by spectrophotometric analysis at 260 nm (NanoDrop Technologies, Wilmington, DE, USA).

### Discovery phase: whole-genome gene expression microarray

**Figure 4:**
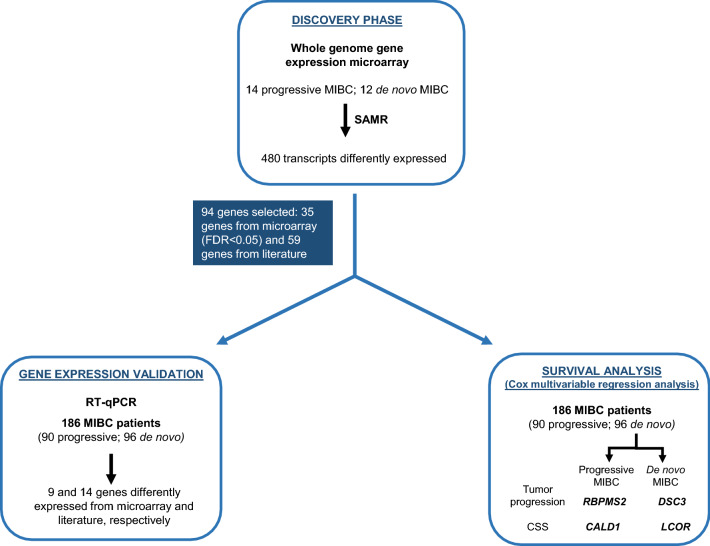
Study outline. Tissue samples were obtained from a total of 104 progressive and 108 de novo MIBC patients. Samples were split into a discovery (26 samples) and validation phase (186 samples). Genes differently expressed between progressive and de novo MIBC patients were identified in the discovery phase by using gene expression microarray. None of the samples from the discovery phase were employed for the validation process. In this validation phase, the differential expression of 94 genes was evaluated. In survival analysis, *RBPMS2* and *DSC3* were identified as prognostic biomarkers for tumor progression in progressive and de novo MIBC, respectively; *CALD1* and *LCOR* were identified as prognostic markers for CSS in progressive and de novo MIBC, respectively. *CSS* Cancer specific survival, *FDR* False discovery rate, *SAMR* Significance analysis of microarrays.

A flowchart of the entire study is shown in Fig. 4. Global gene expression profiling of 26 randomly selected MIBC samples from Hospital Clinic (Table 1), Barcelona, Spain (14 progressive and 12 de novo), was performed by using Whole-Genome Gene Expression DASL HT Assay (Illumina, San Diego, CA, USA) according to manufacturer’s instructions^32^. RNA quality control was performed by quantifying *RPL13A* by reverse transcription quantitative PCR (RT-qPCR) (Applied Biosystems, Foster City, CA, USA), following manufacturer’s instructions. All 26 samples had cycle quantification (Cq) values for *RPL13A* < 28, which are considered to be of acceptable RNA quality by microarray manufacturers (data not shown).

DASL gene expression data was processed employing quantile normalization using the Lumi bioconductor package. Those samples with a 75% of absent probes were discarded. Next, those probes with a coefficient of variation greater than 0.1 were excluded, which resulted in the selection of a total of 27,965 probes from the original set of 29,377. Detection of differentially expressed probes was performed using the SAMR package^33^. Transcripts with a false discovery rate (FDR) < 0.05 were considered as differentially expressed.

R package version 2.0. was used for all calculations and to construct heatmaps (https://CRAN.R-project.org/package=samr). Gene set enrichment analysis (GSEA) was performed using GSEA2-2.2.0 software for testing specific gene set based on Hallmark, Kyoto Encyclopedia of Genes and Genomes (KEGG)^34^ and Reactome pathway databases. Microarray files and clinical information were deposited into Gene Expression Omnibus (GEO) with the accession number: GSE149582 (Supplementary Table S2).

### Validation of microarray expression

Differential expression of 94 genes, 35 genes chosen from microarray analysis (FDR < 0.05) and 59 genes from literature (Supplementary Table S3), was determined in an additional series of 186 patients from Hospital Clinic and Radboud UMC (Table 1) using Biomark 96.96 Dynamic Arrays (Fluidigm, South San Francisco, CA, USA).

Complementary DNA (cDNA) was synthesized from 100 ng of total RNA isolated from MIBC samples using the High-Capacity cDNA Reverse Transcription Kit (Applied Biosystems, Foster City, CA, USA), following manufacturer’s instructions, except that the final volume of the reaction was 25 µl. Each cDNA sample was used for the multiplex pre-amplification using TaqMan PreAmp Master Mix Kit following the manufacturer’s instructions (Applied Biosystems), except that the final volume of the reaction was 5 µl. A total of 94 target genes and 2 endogenous controls (*GUSB* and *PPIA*) were used in a cDNA pre-amplification reaction (Supplementary Table S3). After pre-amplification, quantitative PCR (qPCR) was used to control RNA quality. Samples with *GUSB* Cq values between 18 and 24 were considered of acceptable RNA quality and used for further analysis.

Pre-amplified cDNA and TaqMan Gene Expression Assays 20X (Applied Biosystems) were loaded into the BioMark 96.96 Dynamic Arrays (Fluidigm, South San Francisco, CA, USA) following the manufacturer’s instructions. The real-time qPCR experiments were performed on the Biomark HD system (Fluidigm Corporation).

Fluidigm Real-Time PCR Analysis Software was used to obtain Cq values. Relative expression levels of target genes within a sample were expressed as ΔCq (ΔCq = Cq endogenous control − Cq target gene). The mean Cq value of *GUSB* and *PPIA* was used as endogenous control. Genes with Cq > 34 were considered low expression and were not evaluated. Fold-change values were generated from the median expression of genes from the BioMark 96.96 Dynamic Arrays of groups compared.

Assessment of differential gene expression was performed using the Student’s t test for independent samples. Samples with a *p* value < 0.05 were considered significant. The FDR method was used to correct *p* values for multiple comparisons.

Gene–gene functional interaction network for the differentially expressed genes (DEGs) was built by GeneMANIA Cytoscape 3.6.0 plugin^35^. Co-expression, physical and pathway gene–gene interactions were evaluated. ToppGene (https://toppgene.cchmc.org/)^36^ was used to identify significant pathways for DEGs.

### Survival analysis

Univariate Cox regression analysis was performed on the clinical and molecular variables to examine its influence on tumor progression and CSS. Subsequently, multivariate Cox regression analysis was performed on significant variables. Statistical significance was established at a p-value of 0.05. Gene expression was dichotomized using the median expression value of significant genes. Thereafter, Kaplan–Meier curves were generated.

All analyses were carried out with R-software and SPSS software package (IMB SPSS Statistics 23).

## Supplementary Information

Supplementary Information.

## Abbreviations

AUA: American Urological Association
CSS: Cancer specific survival
Cq: Cycle quantification
DEGs: Differentially expressed genes
EAU: European Association of Urology
EMT: Epithelial-mesenchymal transition
FDR: False discovery rate
FFPE: Formalin-fixed paraffin-embedded
GSEA: Gene set enrichment analysis
KEGG: Kyoto Encyclopedia of Genes and Genomes
LN: Lymph nodes
MIBC: Muscle-invasive bladder cancer
NMIBC: Non-muscle-invasive bladder cancer
RC: Radical cystectomy
RT-qPCR: Reverse transcription quantitative PCR
TCGA: The Cancer Genome Atlas
TURBT: Transurethral resection of the bladder tumor
