# Supplementary material for: Differential gene expression profile between progressive and de novo muscle invasive bladder cancer and its prognostic implication
Source: Sci Rep. 2021 Mar 17;11:6132. doi: 10.1038/s41598-021-85137-1 (PMC7969618; doi:10.1038/s41598-021-85137-1)
Supplement: Supplementary file 1 — Supplementary Information. [file 41598_2021_85137_MOESM1_ESM.pdf]

SUPPLEMENTARY MATERIAL

Differential gene expression profile comparison between progressive and *de novo* muscle invasive bladder cancer: prognostic implications

Raquel Carrasco[1]*, Laura Izquierdo[1]*, Antoine G. van der Heijden[2], Juan José Lozano[3], Marco Franco[1], Mercedes Ingelmo-Torres[1], Fiorella L. Roldan[1], Montserrat Llorens[1], María José Ribal[1], Lourdes Mengual[1], Antonio Alcaraz[1]

[1] *Laboratory and Department of Urology, Hospital Clínic, Institut d'Investigacions Biomèdiques August Pi i Sunyer (IDIBAPS), Universitat de Barcelona, Barcelona, Spain*

[2] *Department of Urology, Radboud University Medical Center, Nijmegen, The Netherlands*

[3] *CIBERehd, Plataforma de Bioinformática, Centro de Investigación Biomédica en red de Enfermedades Hepáticas y Digestivas, Barcelona, Spain*

*These authors have contributed equally to this work

Contact information corresponding author

Lourdes Mengual, Ph.D.
Laboratory of Urology. Hospital Clínic de Barcelona.
Centre de Recerca Biomèdica CELLEX, office B22.
C/Casanova, 143
08036 Barcelona, Spain
Tel: (+34) 93 227 54 00 Ext. 4820
e-mail: lmengual@clinic.cat

Keywords (MeSH): bladder cancer; gene expression profiling; prognosis; *de novo*; progressive

Supplementary Figure S1. Gene-gene interaction network for the genes that define the different gene expression profile of progressive and *de novo* MIBC groups (A) and functional gene enrichment analysis for the genes that define the different gene expression profile of both MIBC groups (B).

A)

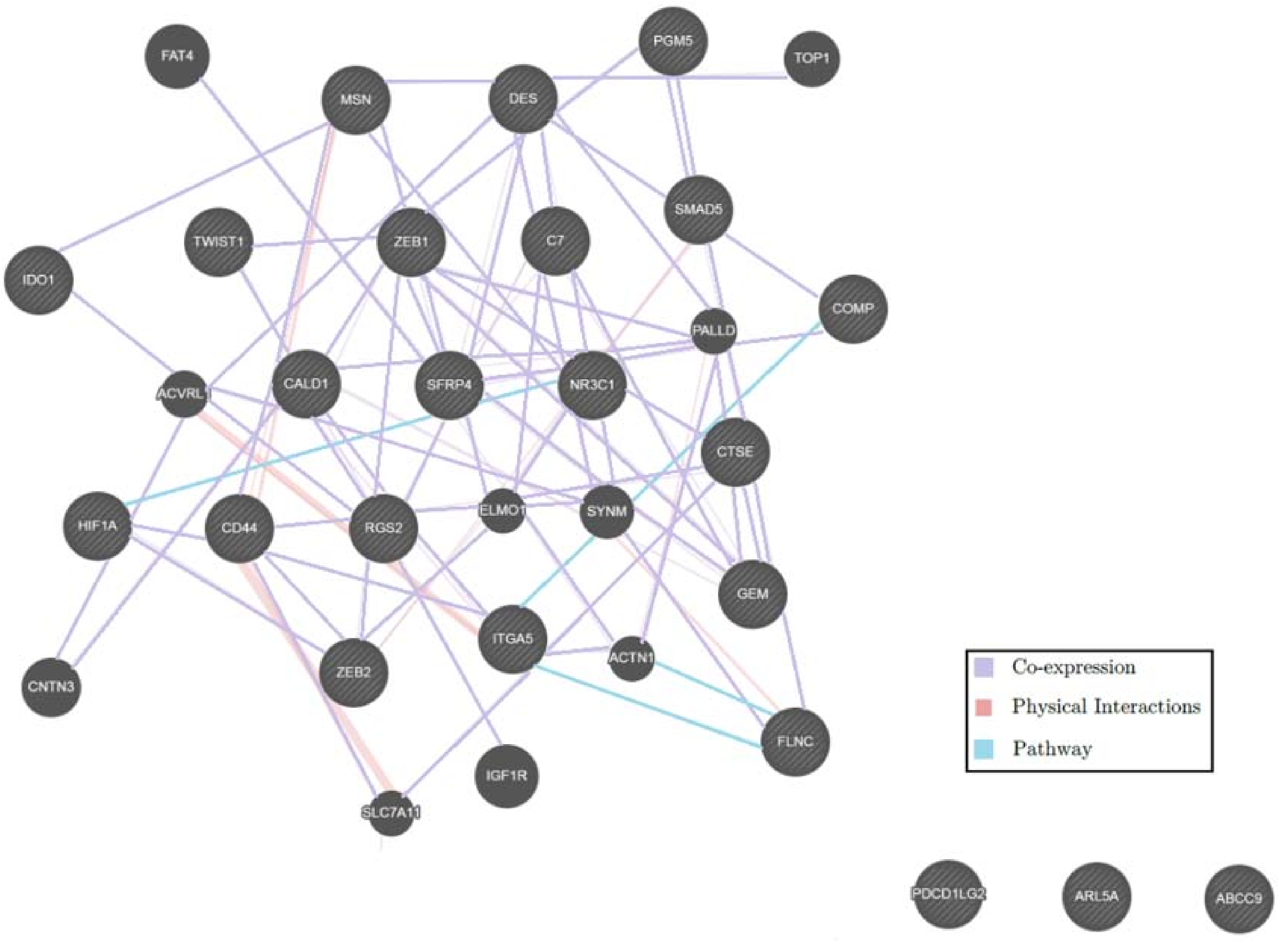

The 23 DEGs between progressive and *de novo* MIBC are shown in striped circles.

B)

ID	NAME PATHWAY	SOURCE	FDR	GENES
782000	*Proteoglycans in cancer	BioSystems: KEGG	<0.001	*CD44, FLNC, HIF1A, TWIST1, ITGA5, MSN*
1383026	*PTK6 Expression	BioSystems: REACTOME	<0.001	*HIF1A, NR3C1*
1270260	Integrin cell surface interactions	BioSystems: REACTOME	<0.001	*CD44, ITGA5, COMP*
83068	*ECM-receptor interaction	BioSystems: KEGG	<0.001	*CD44, ITGA5, COMP*
1470923	Interleukin-4 and 13 signaling	BioSystems: REACTOME	<0.001	*HIF1A, ZEB1, TWIST1*
852705	MicroRNAs in cancer	BioSystems: KEGG	<0.001	*CD44, ZEB2, ZEB1, ITGA5*
1269872	BMAL1: CLOCK, NPAS2 activates circadian gene expression	BioSystems: REACTOME	<0.001	*HIF1A, NR3C1*
169347	Notch-mediated HES/HEY network	BioSystems: Pathway Interaction Database	<0.001	*HIF1A, TWIST1*
83067	*Focal adhesion	BioSystems: KEGG	<0.001	*FLNC, ITGA5, COMP*
1269868	*Muscle contraction	BioSystems: REACTOME	<0.001	*CALD1, DES, ABCC9*
83104	Shigellosis	BioSystems: KEGG	<0.001	*CD44, ITGA5*
1270417	Oxygen-dependent asparagine hydroxylation of Hypoxia-inducible Factor Alpha	BioSystems: REACTOME	<0.001	*HIF1A*
1383025	Signaling by PTK6	BioSystems: REACTOME	<0.001	*HIF1A, NR3C1*
M167	AP-1 transcription factor network	MSigDB C2 BIOCARTA (v7.1)	<0.001	*HIF1A, NR3C1*
1269871	Circadian Clock	BioSystems: REACTOME	<0.001	*HIF1A, NR3C1*
117293	Arrhythmogenic right ventricular cardiomyopathy (ARVC)	BioSystems: KEGG	<0.001	*DES, ITGA5*
1268828	ATP sensitive Potassium channels	BioSystems: REACTOME	<0.001	*ABCC9*
114229	Hypertrophic cardiomyopathy (HCM)	BioSystems: KEGG	<0.001	*DES, ITGA5*
PW:0000330	Bone morphogenetic proteins signaling	Pathway Ontology	<0.001	*SMAD5*
M2642	TGF-beta signaling pathway	MSigDB C2 BIOCARTA (v7.1)	<0.001	*SMAD5, COMP*
M6856	Hematopoietic cell lineage	MSigDB C2 BIOCARTA (v7.1)	<0.001	*CD44, ITGA5*

Abbreviations: FDR; False Discovery Rate

Selected pathways involved in tumor progression are indicated with a *.

Supplementary Figure S2. Kaplan-Meier curve for tumor progression comparing patients having positive or negative lymph nodes (LN).

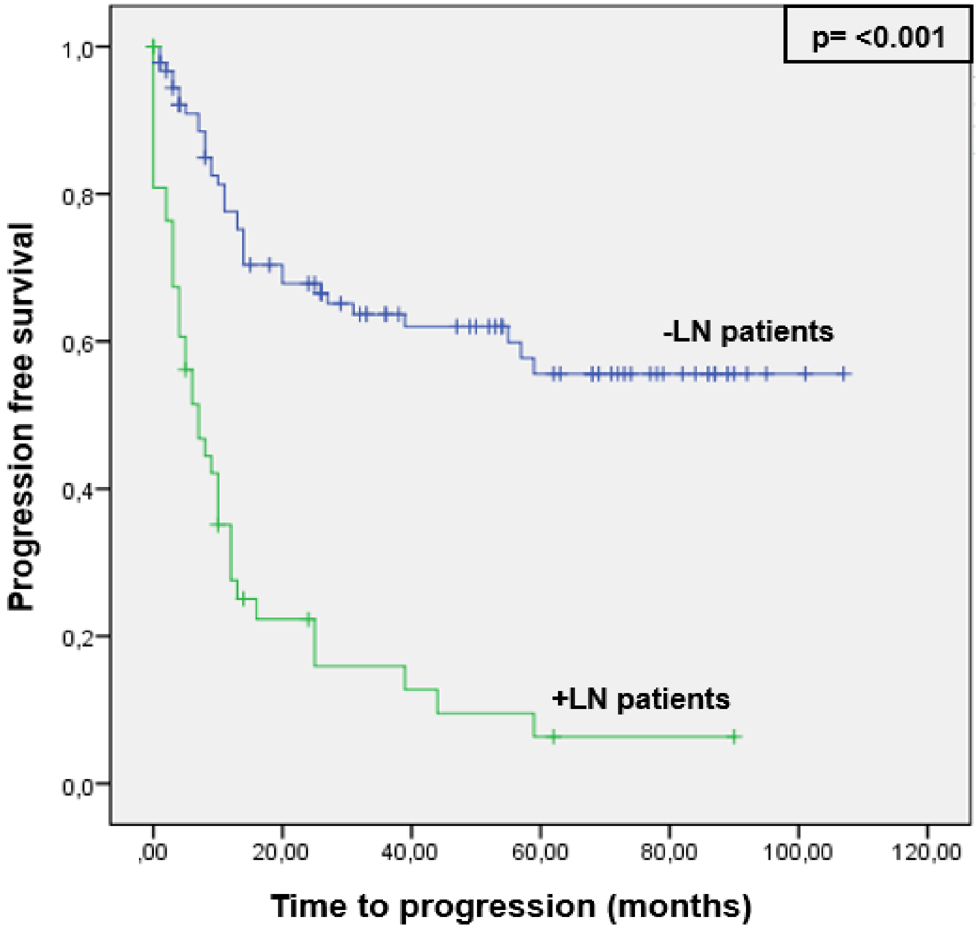

Supplementary Table S1. Differently expressed transcripts between progressive and *de novo* MIBC patients from the discovery phase analyzed by Illumina microarray.

Gene	Fold change	p-Value
SPRR2E	-19,10	<0,001
CALML3	-13,92	<0,001
CEP250	-8,95	<0,001
IFI27	-7,24	<0,001
SFN	-6,17	<0,001
GJB3	-5,69	<0,001
KLHDC7B	-5,60	<0,001
USP42	-5,30	<0,001
TRIM7	-4,87	<0,001
CHMP4B	-4,75	<0,001
CD8A	-3,48	<0,001
EXD3	-3,25	<0,001
FOXF2	1,37	0,003
NARG1L	1,50	0,002
PFN2	1,53	0,002
MTUS1	1,53	0,003
SMAD7	1,55	0,003
ALLC	1,56	0,003
ARL6	1,56	0,002
THAP1	1,56	0,001
UCMA	1,58	0,003
CDC42EP1	1,58	0,002
LSM12	1,59	0,003
AP4S1	1,59	0,003
LOC727811	1,60	0,002
LOC284441	1,61	0,001
CUGBP1	1,62	0,002
FGF2	1,63	0,003
RYK	1,64	0,003
USP46	1,65	0,002
ARAP3	1,67	<0,001
ZNF302	1,68	0,002
CEP57	1,69	0,002
PIGK	1,70	0,001
DNAJC7	1,71	0,003
NEDD9	1,71	0,003
GTF3C6	1,71	0,002
DYNLRB1	1,71	0,002
FNTA	1,72	0,001

SSTR1	1,72	p<0,001
ISCA1L	1,73	0,002
ALK	1,75	0,003
ACCN3	1,75	0,002
FOXP2	1,76	0,002
AQP12B	1,76	0,002
LOC441666	1,76	0,002
NOP2	1,76	0,001
LOC200030	1,77	0,003
TNFSF18	1,77	0,001
MRPL30	1,77	0,001
C13orf33	1,78	0,003
PRKD3	1,80	0,003
MTERF	1,80	0,002
C4BPB	1,81	p<0,001
TMEM59L	1,82	0,001
MMP23B	1,82	0,001
IMPDH1	1,83	0,002
OTUB2	1,84	0,002
UQCRC2	1,84	0,001
STEAP1	1,84	0,003
ZNF32	1,85	0,003
LOC100216545	1,86	0,002
PPP1CB	1,86	0,002
OR7G1	1,86	0,003
ZDHHC9	1,86	0,002
CPOX	1,87	0,003
PHF14	1,87	0,001
RPE	1,87	0,001
TTYH2	1,87	0,001
CBY1	1,87	0,002
ZNF805	1,88	0,001
PIR	1,88	0,001
COPS2	1,89	0,002
C11orf58	1,89	0,003
STAR	1,89	0,003
KRT37	1,89	0,001
ABCA9	1,89	p<0,001
UBE2J2	1,89	0,001
LOC653994	1,89	0,002
KCNH2	1,90	p<0,001
FLJ23584	1,90	0,001
CTRB1	1,91	0,002
ATG4B	1,91	0,002
PPP4R1L	1,91	0,003
NIPSNAP3B	1,91	0,001

PPP2R5C	1,92	0,002
GJC1	1,93	0,001
SNORD58B	1,93	0,002
ZNF557	1,93	0,003
LOC440926	1,93	0,003
PDCD10	1,94	0,002
ALDH1B1	1,94	0,003
ZNF518A	1,94	0,002
NIPBL	1,94	0,002
MOCS1	1,94	0,002
NAP1L1	1,95	0,002
COQ10B	1,95	0,002
G3BP2	1,95	0,002
GRK4	1,97	0,002
CDK10	1,98	0,003
SNORD113 3	1,98	0,002
ACPL2	1,98	0,001
GCOM1	1,98	0,002
RIF1	1,98	0,002
C15orf44	1,99	0,001
SNX9	1,99	0,002
MGC21881	1,99	0,001
RAB20	2,00	0,001
RERE	2,01	0,002
CICK0721Q.1	2,01	p<0,001
CNIH	2,01	0,002
C10orf32	2,02	0,001
TBC1D19	2,02	p<0,001
SCAND2	2,02	0,001
TADA2A	2,02	0,001
C14orf11	2,03	0,001
ECHDC1	2,04	0,001
MAP3K10	2,04	0,002
MYO18A	2,04	0,003
SEC23A	2,05	0,002
DIABLO	2,06	0,002
PHLDA2	2,06	0,003
SLC39A7	2,06	0,002
PLEKHB2	2,06	0,001
ZBTB8A	2,06	0,002
PLAA	2,06	0,002
SERPINA1	2,07	0,003
NCRNA00153	2,07	0,002
TFB2M	2,07	0,001
ACN9	2,09	0,001
TERF1	2,09	0,001

PUS3	2,09	0,002
N4BP2	2,09	0,001
TNPO2	2,09	0,001
NCRNA00095	2,11	0,003
RPSA	2,11	0,001
C7orf70	2,11	0,001
FLJ20125	2,12	0,001
ATAD1	2,12	0,002
ZNF451	2,12	0,001
ZNF321	2,12	0,002
HADHA	2,12	0,001
ZNF706	2,13	0,001
SNORD113 2	2,13	0,001
SLC25A27	2,13	0,002
UBE2MP1	2,13	0,001
CALD1	2,13	0,001
ASB7	2,14	p<0,001
JMJD8	2,15	0,002
CROP	2,15	0,003
ERGIC3	2,15	p<0,001
ZNF703	2,15	0,002
ZFP42	2,16	0,001
ARPP19	2,16	0,001
SLC8A3	2,16	0,001
SLC30A5	2,16	0,001
ANK2	2,16	0,002
NCOA3	2,16	0,001
ZNF493	2,17	0,003
ACSS2	2,17	0,003
SYF2	2,17	0,002
NUP62	2,17	0,002
GADD45A	2,18	0,003
DPP7	2,18	0,002
PPP1R15B	2,18	0,001
BOP1	2,18	0,003
CAMK1	2,18	0,003
FAM63A	2,18	0,002
THEX1	2,19	0,001
LCAT	2,19	0,001
NDFIP1	2,19	0,002
SFXN4	2,20	0,001
MARCH5	2,20	0,001
MBNL3	2,20	0,002
FXYD1	2,21	0,002
MMP16	2,21	p<0,001
MXRA7	2,21	p<0,001

SCOC	2,22	0,003
NKAPL	2,22	0,001
HOXC6	2,22	0,001
EDNRA	2,22	0,003
ESF1	2,22	0,001
MAGOHB	2,22	0,001
RPS26L	2,23	0,001
LONP2	2,23	0,002
HOXA10	2,23	0,002
PTH1R	2,23	0,001
ZCCHC10	2,24	0,002
HSP90B3P	2,24	0,002
FAM108B1	2,24	0,001
EFCAB2	2,25	0,001
PLN	2,25	0,001
LYSMD3	2,26	0,001
COX19	2,27	0,001
MYADM	2,27	p<0,001
CCDC104	2,27	0,001
C18orf32	2,27	0,002
ALDH4A1	2,28	0,003
C1GALT1C1	2,28	0,000
BMP8B	2,28	0,002
PTX3	2,29	p<0,001
RFX4	2,29	0,001
KIAA1683	2,29	p<0,001
ZNF777	2,29	p<0,001
CDC37L1	2,29	0,003
COX18	2,30	0,002
HPS1	2,30	0,003
BCL6B	2,30	0,001
NOC3L	2,30	0,002
PREPL	2,30	0,001
NLRP12	2,31	p<0,001
MOCS1	2,31	p<0,001
GPBP1L1	2,31	0,002
CHRDL1	2,31	0,002
SLC36A4	2,31	0,002
KLHL8	2,32	p<0,001
PDLIM3	2,32	0,003
TMEM44	2,32	0,003
C21orf130	2,33	0,003
ACTR1A	2,33	0,001
BGN	2,33	p<0,001
GPR116	2,34	0,002
RBM41	2,34	0,002

MOBKL1A	2,34	0,001
PTPLB	2,34	0,001
TMEM44	2,34	0,002
C15orf58	2,35	0,001
DDX52	2,35	p<0,001
ANKS6	2,35	0,002
FBLN1	2,35	0,002
ISYNA1	2,36	p<0,001
DENND4C	2,36	0,001
NCLN	2,36	0,002
NR1H3	2,36	0,002
TMEM183A	2,36	p<0,001
PPIE	2,37	0,001
ST7	2,37	0,001
ZNF358	2,38	p<0,001
KLHDC10	2,38	p<0,001
PSIP1	2,38	0,001
WASF1	2,39	0,002
ATG3	2,39	p<0,001
RAB27B	2,39	0,002
TEAD3	2,40	0,001
CHD9	2,40	0,001
PSMC3IP	2,40	0,001
RSRC2	2,40	0,002
RPS7	2,41	p<0,001
PXDN	2,41	0,001
PPP1R12B	2,41	0,001
UGP2	2,41	0,002
FBXO38	2,42	0,002
IDS	2,42	0,001
SCARA3	2,42	0,001
NUCKS1	2,42	0,001
CNBP	2,42	0,001
NR4A3	2,44	0,003
SFXN3	2,44	0,002
GPX8	2,44	0,002
ADRB3	2,44	0,001
FGFR1	2,44	0,002
SLC30A5	2,44	p<0,001
PCDHGA9	2,45	0,001
CXCL6	2,45	p<0,001
NR4A3	2,45	0,002
RFWD2	2,45	p<0,001
FAM26E	2,45	p<0,001
TRAPPC6B	2,46	0,002
TLN1	2,46	0,003

TLK1	2,46	0,003
NAMPT	2,46	0,001
ARL5A	2,47	0,001
RDH14	2,48	0,001
TMEM183B	2,49	p<0,001
GPR162	2,49	0,003
KIAA0090	2,50	0,002
NBLA00301	2,51	0,003
ZBTB1	2,51	0,002
RBM9	2,51	p<0,001
AP1S2	2,51	0,002
SLMO1	2,51	p<0,001
SERPINE1	2,51	0,001
NFE2L2	2,51	0,001
C7orf41	2,52	0,002
DUSP6	2,53	0,002
C21orf2	2,53	0,001
KCTD1	2,54	0,001
DDX20	2,55	p<0,001
MYLK	2,55	0,002
INO80B	2,55	0,001
METTL2B	2,57	p<0,001
TM9SF3	2,57	0,001
BHMT2	2,57	0,002
NELF	2,57	0,002
C1orf123	2,57	p<0,001
SON	2,57	0,001
PBLD	2,57	p<0,001
LPAR4	2,58	0,001
LMO3	2,58	p<0,001
ITM2A	2,58	p<0,001
EIF4E	2,58	p<0,001
COL4A2	2,58	0,001
DLC1	2,59	0,002
SLC35B4	2,59	p<0,001
SLC30A7	2,59	0,001
HCFC1R1	2,59	p<0,001
SKAP2	2,60	0,001
GlYD2	2,60	0,002
PRND	2,60	0,001
C10orf119	2,60	0,002
TSC22D3	2,61	0,001
GAB2	2,61	p<0,001
C4orf27	2,61	p<0,001
ARHGAP21	2,62	0,003
ASB1	2,62	p<0,001

ABTB1	2,62	p<0,001
DUSP3	2,63	p<0,001
CLIP3	2,63	0,001
ACOT2	2,64	p<0,001
RERG	2,64	0,001
SERAC1	2,64	p<0,001
AXUD1	2,65	0,002
CCNG1	2,65	0,001
FASTK	2,65	p<0,001
SPAG16	2,66	0,001
FAS	2,66	p<0,001
VPS24	2,66	0,003
ECM2	2,66	0,000
C10orf10	2,66	0,001
C9orf82	2,67	p<0,001
RAP1B	2,67	0,001
DCAF10	2,67	0,001
PGAM4	2,68	0,002
LAMB1	2,68	p<0,001
ZNF542	2,69	p<0,001
ASB3	2,69	p<0,001
MID1	2,69	0,001
KTELC1	2,69	p<0,001
SUZ12	2,70	p<0,001
CDH2	2,70	0,001
C4orf18	2,71	0,001
ARL4A	2,71	p<0,001
PPAP2B	2,71	0,001
THBS3	2,73	0,001
ANXA11	2,73	0,001
C21orf33	2,74	0,002
FLJ21986	2,74	0,002
INVS	2,74	p<0,001
C1D	2,74	0,001
GPER	2,74	0,003
EEF1E1	2,75	p<0,001
CXCL2	2,76	0,001
AGL	2,76	0,003
FAM103A1	2,76	0,001
SERINC3	2,77	0,002
SLC12A2	2,77	0,002
KCNT2	2,78	p<0,001
SR140	2,78	p<0,001
PPP2R3A	2,79	p<0,001
NNT	2,79	p<0,001
NME1	2,79	p<0,001

ACACB	2,81	0,001
BAG2	2,82	p<0,001
SMAD5	2,82	0,001
FLJ22795	2,83	p<0,001
PANX2	2,83	0,001
PTGIS	2,83	0,003
SLC35C2	2,84	p<0,001
NUPL1	2,85	0,001
ELANE	2,86	0,002
CSNK1G2	2,87	0,001
SORBS1	2,87	0,002
SFRS5	2,87	0,001
TSPAN4	2,88	0,002
FANCL	2,88	0,001
NME2	2,88	0,003
C9orf37	2,90	0,002
BOLA2	2,90	0,001
SMAD9	2,92	p<0,001
ARL6IP5	2,92	0,002
C15orf5	2,92	p<0,001
ADAM22	2,93	p<0,001
LIMS1	2,94	0,001
CFLAR	2,96	0,001
DEGS1	2,99	0,001
DNAJB14	3,00	p<0,001
LEPR	3,01	0,001
CSPG4	3,01	0,001
C1orf215	3,03	0,003
RPS6KA3	3,03	p<0,001
METTL1	3,03	0,002
CBX3	3,03	p<0,001
SYNPO2	3,06	0,001
ICK	3,06	p<0,001
GEM	3,06	0,003
NLF2	3,06	0,002
KCNMA1	3,06	p<0,001
FAM43A	3,10	0,002
PDF	3,11	p<0,001
ZNF644	3,11	0,001
BBS5	3,12	p<0,001
DSTN	3,12	p<0,001
STXBP6	3,13	p<0,001
PEAR1	3,13	p<0,001
SMTN	3,14	0,001
LAMA4	3,15	p<0,001
RANGRF	3,15	p<0,001

DNAJC25		
GNG10	3,16	p<0,001
XLKD1	3,16	0,002
RDH11	3,17	p<0,001
TAF9B	3,17	p<0,001
SPRY2	3,20	0,001
TCEA2	3,22	0,003
TAGLN	3,23	0,001
NR4A2	3,23	0,001
GCNT1	3,25	0,002
ZYG11B	3,25	p<0,001
ZNF521	3,25	0,001
FERMT2	3,26	0,001
GHR	3,29	p<0,001
HIF1A	3,30	p<0,001
C21orf34	3,32	0,002
SEPT11	3,35	0,001
FABP4	3,37	0,003
DACT3	3,38	p<0,001
RNF150	3,39	p<0,001
ALDH5A1	3,39	p<0,001
DIO3OS	3,40	0,003
SYNE1	3,40	p<0,001
LOC200030	3,42	p<0,001
CD55	3,43	p<0,001
GABBR1	3,43	p<0,001
CILP	3,45	0,001
ADAMTSL4	3,48	0,003
C6orf62	3,49	p<0,001
TAF1B	3,50	p<0,001
CILP	3,54	0,001
FAM108A1	3,54	p<0,001
NNMT	3,57	p<0,001
GSTM1	3,58	p<0,001
LOC401720	3,59	p<0,001
RRM2B	3,61	p<0,001
CSDC2	3,61	0,002
RASD2	3,62	0,002
LCOR	3,63	p<0,001
NDUFA5	3,67	p<0,001
IL6	3,67	0,002
FAM133B	3,68	p<0,001
FHL2	3,69	p<0,001
PLCD4	3,69	0,001
SKP1A	3,73	p<0,001
CA3	3,76	p<0,001

RBPMS2	3,82	p<0,001
GEM	3,84	p<0,001
HTR2A	3,86	p<0,001
PHF20L1	3,89	p<0,001
PTGS2	3,89	0,001
CALD1	3,90	p<0,001
PCP4	3,98	p<0,001
ABCC9	4,10	p<0,001
SELT	4,12	p<0,001
RGS11	4,21	p<0,001
PDE1A	4,22	p<0,001
RAB33B	4,23	p<0,001
RGS2	4,25	0,001
ITGA5	4,26	p<0,001
GTF2A1L	4,27	p<0,001
CYB5R1	4,37	p<0,001
INHBA	4,37	p<0,001
CYP19A1	4,46	p<0,001
ADAMTS4	4,77	p<0,001
FLNC	4,83	0,001
RPESP	5,05	p<0,001
DSEL	5,27	p<0,001
AKR1C1	5,30	p<0,001
KGFLP1	5,44	p<0,001
INS IGF2	5,50	p<0,001
GSTM1	9,28	p<0,001
GSTM2	9,75	p<0,001
FOSB	10,18	p<0,001

Supplementary Table S3. Clinical annotations for the cohort of discovery phase (corresponding to GEO GSE149582 dataset).

	LAB CODE	SEX	AGE (years)	Type of surgery	pT	Grade	Node	NAC	CIS	PROGRESSION	TIME TO PROGRESSION (months)	OVERALL SURVIVAL	TIME TO DEAD (months)	CANCER SPECIFIC SURVIVAL
DE NOVO MIBC	B778	M	56	TURB	T2	HG	N0	1	1	1	35	1	58	1
	B1202	M	48	TURB	T2	HG	N0	1	0	0	98	0	-	0
	B1192	M	58	TURB	T2	HG	N0	1	1	NA	NA	NA	NA	NA
	B897	M	73	TURB	T2	HG	N0	1	1	0	73	1	73	0
	B643	M	63	TURB	T2	HG	N0	1	0	0	89	0	-	0
	B1216	M	70	TURB	T2	HG	N0	1	1	0	73	0	-	0
	B1218	M	68	TURB	T2	HG	N0	1	1	0	73	0	-	0
	B1197	M	53	TURB	T2	HG	N0	1	1	0	115	0	-	0
	B1193	M	70	TURB	T2	HG	N1	1	1	1	5	1	8	1
	B1196	M	70	TURB	T2	HG	N2	1	0	1	5	1	7	1
	B1776	M	84	Cystectomy	T2	HG	N0	0	0	0	91	1	91	0
	B1830	M	79	Cystectomy	T4	HG	N1	0	0	1	5	1	6	1
PROGRESSIVE MIBC	B155	F	82	Cystectomy	T2	HG	N0	0	0	1	11	1	12	1
	B336	M	77	Cystectomy	T2	HG	N0	0	0	0	92	0	-	0
	B1681b	M	70	Cystectomy	T2	HG	N0	0	0	1	4	1	4	1
	B1682b	M	87	Cystectomy	T2	HG	N0	0	0	0	0	1	0	0
	B1683b	M	72	Cystectomy	T1	HG	N0	0	0	1	69	1	76	1
	B1684b	M	68	Cystectomy	T2	HG	N0	0	0	0	1	1	1	0
	B1691	M	79	TURB	T2	HG	NA			1	24	1	24	1
	B1846	M	69	Cystectomy	T2	HG	N0	0	1	1	15	1	37	1
	B1848	M	54	Cystectomy	T3	HG	N0	0	0	0	2	1	2	0
	B1850	M	79	Cystectomy	T3	HG	N0	0	1	1	19	1	21	1
	B1852	M	74	Cystectomy	T4	HG	N0	0	0	1	1	1	11	1
	B1866	M	79	Cystectomy	T2	HG	N0	0	0	0	0	1	0	0
	B1868	M	71	Cystectomy	T2	HG	N0	0	1	0	73	1	73	0
	B688	M	78	Cystectomy	T3	HG	N0	0	0	0	9	1	9	0

LEGEND

0/1	NO/YES
HG	High grade
TURB	Transurethral resection of the bladder
SEX M/F	Male/Female
NAC	Neoadjuvant chemotherapy
NA	Non available

Supplementary Table S3. Genes analyzed in Biomark 96.96 Dynamic Arrays (Fluidigm).

GENES FROM TCGA[1] (N= 52)		GENES IDENTIFIED BY MICROARRAY EXPERIMENT (N= 33)	GENES INVOLVED IN IMMUNE AND PROGRESSION MECHANISMS[2-10] (N=9)	ENDOGENOUS GENES (N= 2)
Luminal markers	*KRT20*	*ABCC9*	*PDCD1*	*BGUS*
	PPARG	*ARL5A*	*CTLA4*	*PPIA*
	FOXA1	*CALD1*	*CD80*	
	GATA3	*CHMP4B*	*AR*	
	SNX31	*CYB5R1*	*FOXM1*	
	UPK1A	*DSEL*	*ERBB2*	
	UPK2	*DUSP3*	*EGFR*	
	FGFR3	*DUSP6*	*IGF2*	
ECM and smooth muscle	*PGM5*	*FANCL*	*STAT3*	
	DES	*FLNC*		
	C7	*FOSB*		
	SFRP4	*GEM*		
	COMP	*GSTM2*		
	SGCD	*HIF1A*		
EMT and Claudin markers	*ZEB1*	*HTR2A*		
	ZEB2	*IFI27*		
	SNAI1	*INS-IGF2*		
	TWIST1	*LAMB1*		
	CDH2	*METTL1*		
	CLDN7	*PLCD4*		
	CLDN4	*PTGS2*		
Basal markers	*CD44*	*RAB33B*		
	KRT6A	*RGS2*		
	KRT5	*FGFR1*		
	KRT14	*RSRC2*		
	COL17A1	*SELT*		
Squamous markers	*DSC3*	*S EPT 11*		
	GSDMC	*RBPMS2*		
	TGM1	*LCOR*		
	PI3	*SMAD5*		
	TP63	*SPRY2*		
Neuronal-differentiation	*MSI1*	*USP42*		
	PLEKHG4B	*ITGA5*		
	GNG4			
	PEG10			
	RND2			
	APLP1			
	SOX2			
	TUBB2B			
Immune markers	*CD274*			
	PDCD1LG2			
	IDO1			
	CXCL11			
	L1CAM			
	SAA1			
Down-regulated in CIS	*CRTAC1*			
	CTSE			
	PADI3			
Up-regulated in CIS	*MSN*			

	NR3C1
Sonic Hedgehog	*SHH*
	BMP5

Thirty-three of 94 genes were selected because of their differential expression (FDR<0.05) in microarray experiments, 52 genes were selected from The Cancer Genome Atlas (TCGA)[1] and 9 genes were selected from the literature[2–10] because of their involvement in immune and progression mechanisms in MIBC.

1. Robertson, A. G. *et al.* Comprehensive Molecular Characterization of Muscle-Invasive Bladder Cancer. *Cell.* **174**, 1033 (2018).

2. Zichi, C. *et al.* Immunotherapy for Patients with Advanced Urothelial Cancer: Current Evidence and Future Perspectives. *Biomed Res. Int.* **2017**, 5618174 (2017).

3. Ali, A. *et al.* Prognostic significance of epidermal growth factor receptor (EGFR) over expression in urothelial carcinoma of urinary bladder. *BMC Urol*. **18**, 59 (2018).

4. Jitao, W. *et al.* Androgen receptor inducing bladder cancer progression by promoting an epithelial – mesenchymal transition. *Andrología*. **46**, 1128-1133 (2014).

5. The Cancer Genome Atlas *et al.* Comprehensive molecular characterization of urothelial bladder carcinoma. *Nature.* **507**, 315-322 (2014).

6. Koga, F., Takemura, K. & Fukushima, H. Biomarkers for Predicting Clinical Outcomes of Chemoradiation-Based Bladder Preservation Therapy for Muscle-Invasive Bladder Cancer. *Int. J. Mol. Sci.* **19**, 2777 (2018).

7. Rinaldetti, S. *et al.* FOXM1 predicts disease progression in non-muscle invasive bladder cancer. *J. Cancer Res. Clin. Oncol.* **144**, 1701–1709 (2018).

8. Tomlinson, D. C., Baxter, E. W., Loadman, P. M., Hull, M. A. & Knowles, M. A.

FGFR1-Induced Epithelial to Mesenchymal Transition through MAPK / PLC c / COX-2-Mediated Mechanisms. *PLoS One*. **7,** e38972 (2012).

9. Kamoun, A. *et al.* The consensus molecular classification of muscle-invasive bladder cancer. *Eur. Urol.* **77**, 420-433 (2020).

10. Roudnicky, F. *et al.* High expression of insulin receptor on tumour-associated blood vessels in invasive bladder cancer predicts poor overall and progression-free survival. *J. Pathol.* **242**, 193–205 (2017).
